# Delineation of the central melanocortin circuitry controlling the kidneys by a virally mediated transsynaptic tracing study in transgenic mouse model

**DOI:** 10.18632/oncotarget.11956

**Published:** 2016-09-10

**Authors:** Tao Tao Liu, Bao Wen Liu, Zhi Gang He, Li Feng, San Guang Liu, Hong Bing Xiang

**Affiliations:** ^1^ Department of Anesthesiology and Pain Medicine, Tongji Hospital of Tongji Medical College, Huazhong University of Science and Technology, Wuhan, Hubei, People's Republic of China; ^2^ Department of Hepatobiliary Surgery, The Second Hospital, Hebei Medical University, Shijiazhuang, People's Republic of China

**Keywords:** central melanocortin circuitry, kidney, pseudorabies virus, neurochemical phenotype, melanocortin-4 receptor, Pathology Section

## Abstract

To examine if brain neurons involved in the efferent control of the kidneys possess melanocortin-4 receptor (MC4-R) and/or tryptophan hydroxylase (TPH). Retrograde tracing pseudorabies virus (PRV)-614 was injected into the kidneys in adult male MC4R-green fluorescent protein (GFP) transgenic mice. After a survival time of 3-7 days, spinal cord and brain were removed and sectioned, and processed for PRV-614 visualization. The neurochemical phenotype of PRV-614-positive neurons was identified using double or triple immunocytochemical labeling against PRV-614, MC4R, or TPH. Double and triple labeling was quantified using microscopy. The majority of PRV-614 immunopositive neurons which also expressed immunoreactivity for MC4R were located in the ipsilateral intermediolateral cell column (IML) of the thoracic spinal cord, the paraventricular nucleus (PVN) of the hypothalamus, and raphe pallidus (RPa), nucleus raphe magnus (NRM) and ventromedial medulla (VMM) of the brainstem. Triple-labeled MC4R/PRV-614/TPH neurons were concentrated in the PVN, RPa, NRM and VMM. These data strongly suggest that central MC4R and TPH are involved in the efferent neuronal control of the kidneys.

## INTRODUCTION

As an important determinant of balancing the volume and composition of body fluids, the kidneys play a critical role in the homeostatic control of water and sodium [1–3], and understanding the mechanisms of their central control could lead to pharmacological and behavioral therapeutic tools of renal-related disease, e.g. renal hypertension and renal diabetes. It is clear that kidneys are received from the body in the form of circulating hormones (arginine vasopressin (AVP), angiotensin AT1, etc.) [4–7], fuels (glucose, etc.) [8, 9], and other factors from the internal or external stimuli (chronic cold exposure, plasma volume expansion, etc.) [10, 11]. Activation of renal sympathetic nerves produces marked changes in renal hemodynamics, tubular ion, water transport and renin secretion [12, 13]. In addition, many other sensory, metabolic, and emotional factors also influence renal sympathetic activity or induce the change of renal function, suggesting that between brain and kidneys comprise a complex neural circuit that integrates multiple signals relevant for this change.

A large number of significant scientific reports demonstrated that the melanocortinergic signaling pathway constitutes a major signaling system in the control of energy homeostasis [14–27]. It has been evidenced that the central melanocortin system plays an important role in the regulation of basal plasma insulin levels and glucose tolerance [28–32]. A growing body of literature supports that sympathetic activity are tightly interconnected *via* hypothalamic melanocortinergic pathways involving the melanocortin-4 receptor (MC4R), a G protein-coupled, seven-transmembrane receptor expressed in the brain [15, 33–35]. MC4R-knockout mice, and loss-of-function mutations in MC4R in humans, are associated with severe obesity and hyperinsulinemia, supporting an essential role for the melanocortin system in the regulation of glucose metabolism [36, 37]. It was shown that the renal sympathoexcitatory responses to leptin and insulin are dependent on the MC4R, suggesting an important role for the MC4R in the regulation of renal sympathetic nerve outflow [38]. In addition, MC4R is found in numerous brain nuclei [33, 37, 39–42], including the sites known to be in the autonomic circuitry of the kidney, such as the hypothalamic paraventricular nucleus (PVH), the dorsomedial hypothalamus (DMH), the raphe pallidus (RPa) of the brainstem, etc [43–45]. However, the exact neurosubstrate underlying the regulation of renal function by the central melanocortin system has not been well defined. The main objective of this study is to provide direct neuroanatomical evidence for the central melanocortin circuits connecting to renal tissues via autonomic nervous system.

## RESULTS

### Time course of multisynaptic CNS projections to the kidney

The entire spinal cord and brain were examined for PRV-614 and MC4R-GFP immunoreactivity. Viral immunoreactive labeling was monitored at 1-day intervals, up to 6 days after renal injection. Postinjection intervals shorter than 3 days (1~2 day) did not result in viral labeling of any neurons in the CNS.

**Figure 1:**
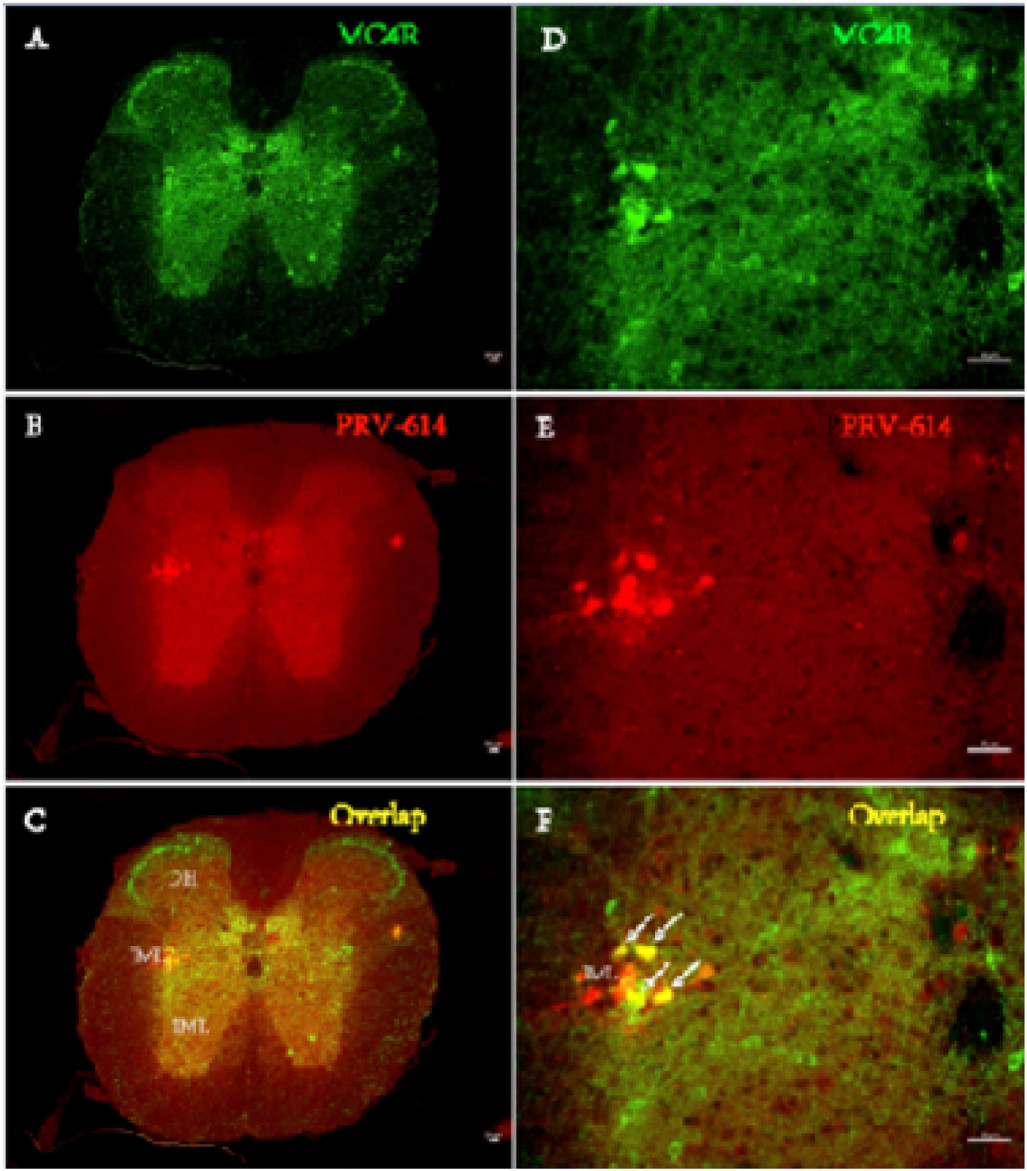
Fluorphor expression illustrating the distributions of PRV-614 (red) and MC4R (green) in transverse section of spinal cord IML in MC4R-GFP transgenic mice Infected sympathetic preganglionic neurons (SPN) neurons in the thoracic IML 4 days after injection of PRV-614 into the kidneys are illustrated. **A.** MC4R-GFP expressing neurons; **B.** PRV-614 expressing neurons in same section as (A); **C.** overlap of (A) and (B), depicting distribution of MC4R-GFP-IR and PRV-614-bearing neurons (while arrow). **D.**, **E.** and **F.**, amplified views of (A, B and C), respectively. IML, the intermediolateral cell column; IC intercalates nucleus; CAN, central autonomic nucleus; DH, Dorsal horn; VH, ventral horn. Scale bar: 50μm.

After 3 days of renal PRV-614 infection (*n* = 3, phase I), colocalization of PRV-614 and MC4R-GFP immunopositive neurons was seen and largely confined to areas in the sympathetic preganglionic neurons (SPNs) in the ipsilateral intermediolateral cell column (IML) of the thoracic spinal cord (Figure 1). Additionally, the kidneys of two mice began to show colocalization of PRV-614 and MC4R-GFP in the paraventricular nucleus of the hypothalamus (PVN).

**Figure 2:**
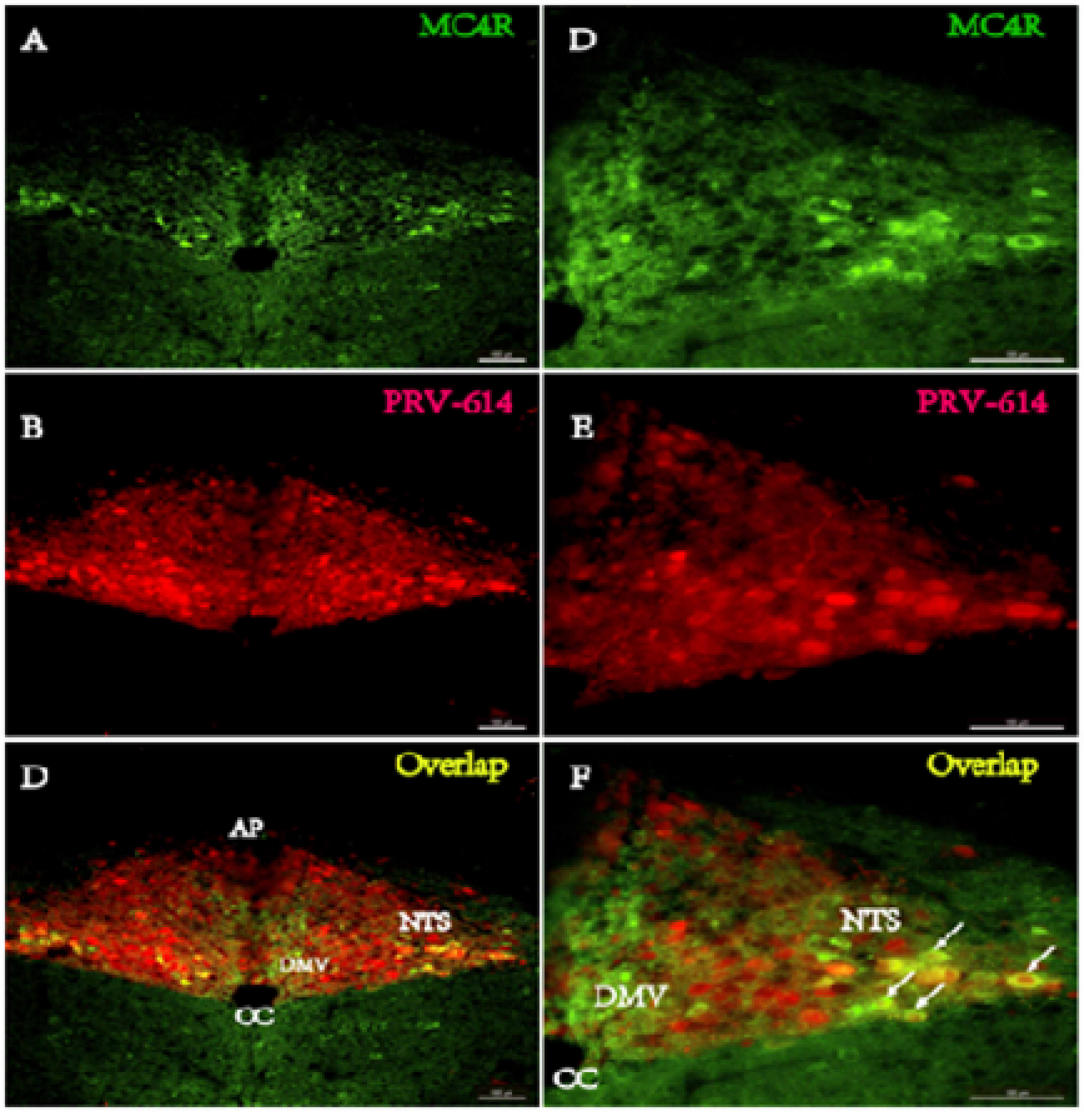
Fluorphor expression illustrating the distributions of PRV-614 (red) and MC4R (green) in the caudal brainstem in MC4R-GFP transgenic mice **A.**-**C.** Low magnification images of a section from the caudal brainstem obtained 4 d after the kidney was injected with PRV-614-ERFP (A, red) and labeled with MC4R-GFP (B, green). C). The images in A and B have been merged with an image showing NTS neurons retrogradely labeled by intra-kidney injection of PRV-614-ERFP. Note that viral labeling is absent in the DMV and restricted to a subset of neurons in the known location of NTS. **D.**-**E.** High magnification images of the region in panel C, to show the overlap of the viral label (D) and the MC4R-GFP (E) in NTS neurons. F). Merged image of D and E. Scale bars: 50 μm. AP, area postrema; CC, Central canal; DMV, dorsal motor nucleus of the vagus; NTS, Nucleus of the solitary tract.

At 4 days after kidney injection (*n* = 3, phase II), PRV-614 immunopositive neurons were detected in the brainstem (e.g., rostral ventrolateral medulla (RVLM), A5 noradrenergic cell region (A5) and nucleus of the solitary tract (NTS), Figure 2) and the paraventricular nucleus of the hypothalamus (PVN) that give rise to direct descending projections to IML of the thoracic spinal cord. The majority of MC4R-GFP/PRV-614 double-labeled neurons were found in the PVN, raphe nuclei (RPa) and NTS, and with a less extent in the RVLM and A5.

**Figure 3:**
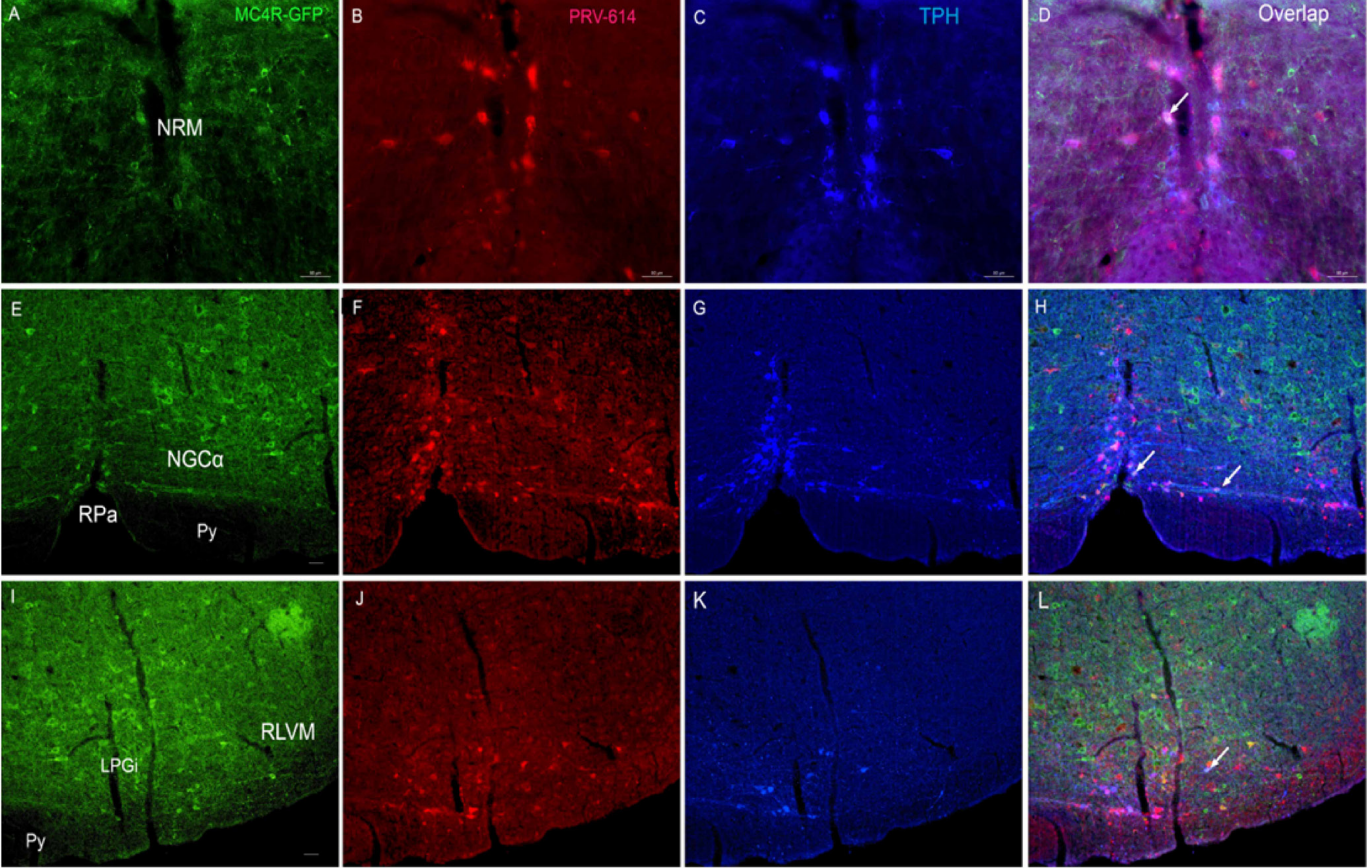
Triple fluorphor expression in the ventral brainstem 5 days after renal PRV-614 injection of MC4R-GFP transgenic mice **D.** Distributions of MC4R-GFP- (**A.**, green), PRV-614 (**B.**, red) and TPH-IR-cells (**C.**, blue) in the NRM 5 days after renal PRV-614 injection; **H.** Distributions of MC4R-GFP- (**E.**, green), PRV-614 (**F.**, red) and TPH-IR-cells (**G.**, blue) in the RPa and NGCα 5 days after renal PRV-614 injection; L) Distributions of MC4R-GFP-(**I.**, green), PRV-614-(**J.**, red) and TPH-IR-cells (**K.**, blue) in the LPGi 5 days after renal PRV-614 injection. Triple-labeled cells were indicated by white arrows. Scale bar: A-D for 50 μm; E-H for 25 μm; I-L for 50 μm. LPGi, lateral gigantocellular reticular nucleus; NGCα, gigantocellular reticular nucleus α part; NRM, nucleus raphe magnus; Py, pyramidal tract; RLVM, lateral ventromedial medulla; RPa, the raphe pallidus.

**Figure 4:**
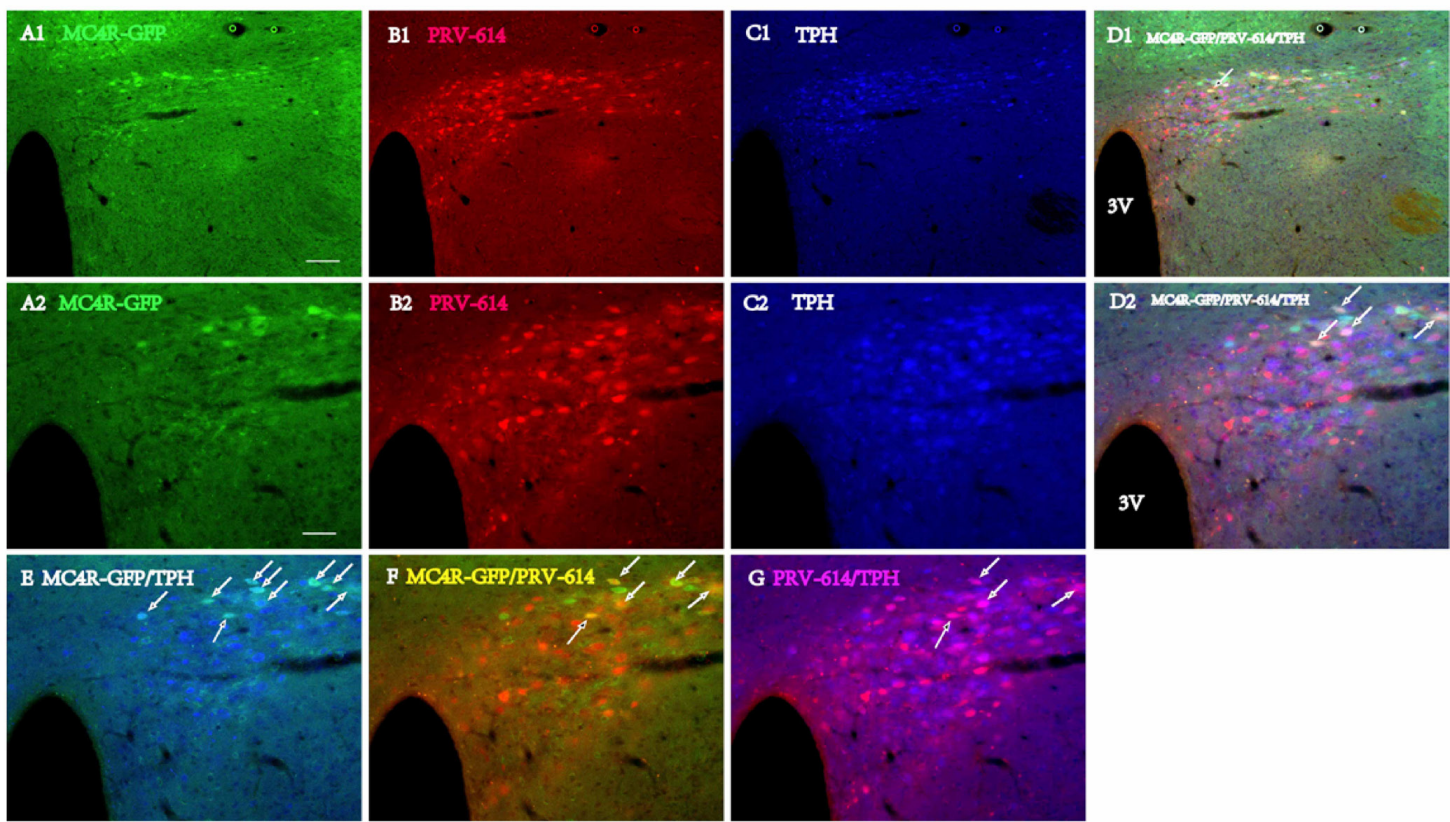
Triple fluorophor Expression in Diencephalon and the paraventricular hypothalamic nucleus (PVN) 6 days after renal PRV-614 injection of MC4R-GFP transgenic mice **A1.** MC4R-GFP neurons (green) in the PVN, **B1.** same section as (A1), and the distribution of PRV-614 infected neurons in diencephalon at the level of the PVN is illustrated. **D1.** Distributions of MC4R-GFP- (A1, green), PRV-614 (B1, red) and TPH-IR-cells (**C1.**, blue) in the PVN 6 days after renal PRV-614 injection; **A2.** A higher magnification image of (A1) showing MC4R-bearing neurons in the RPa (green), **B2.** Same section as (A2), depicting PRV-614-infected neurons (red), **C2.** Same section as (A2), depicting TPH positive neurons (blue), **D2.** Same section as (A2), and triple-labeled cells were indicated by white arrows. **E.** Overlap of (A2) and (C2), depicting the neurons that co-express PRV-614 and TPH (cyan, with white arrows). **F.** Overlap of (A2) and (B2), depicting the neurons that co-express PRV-614 and MC4R-GFP (yellow, with white arrows). **G.** Overlap of (B2) and (C2), depicting the neurons that co-express PRV-614 and TPH (pink, with white arrows). Scale bar: A1-D1 for 50 μm; A2-D2, E-G for 100 μm.

**Figure 5:**
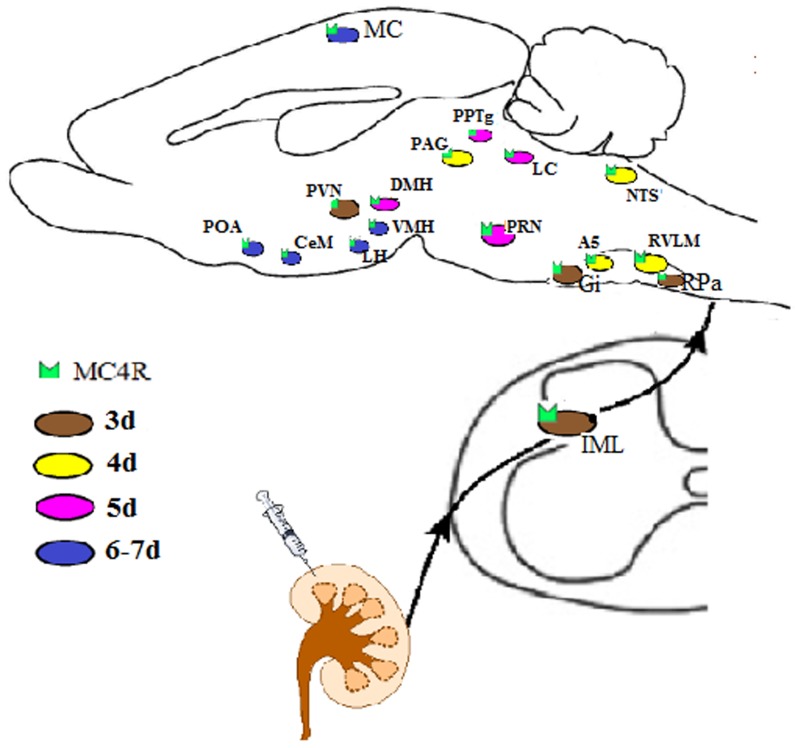
Schematic diagram showing MC4R-expressing areas in the mouse brain infected at different intervals after PRV-614 injection into kidney (brown, 3d; yellow, 4d; purple, 5d; blue, 6-7d), suggesting that MC4R signaling in the neural circuitry controlling the kidneys mediated by sympathetic pathway A5, A5 noradrenergic cell region; CeM, central nucleus of the amygdala; DMH, dorsomedial nucleus of the hypothalamus; Gi, gigantocellular reticular nucleus; IML, intermediolateral cell column;LC, Locus ceruleus; LH, lateral hypothalamus; LPB, lateral parabrachial nucleus; LTD, laterodorsal tegmental nucleus; MC, motor cortex; MC4R, melanocortin4receptor; NTS, nucleus of the solitary tract; PAG, periaqueductal gray; PRN, pontine reticular nucleus; POA, preoptic area; PPTg, pedunculopontine tegmental nucleus; PVN, paraventricular nucleus of the hypothalamus; RPa, raphe pallidus; VMH, ventromedial hypothalamic nucleus.

At 5 days postinjection (*n* = 4, phase III), the number of PRV-614 infected neurons in regions shown at 4 days was increased and the infection spread transneuronally to neurons in other regions of the medulla (e.g., nuclei raphes, Figure 3, 4, rostral ventromedial medulla (RVMM), Figure 3, 4, locus coeruleus (LC)), midbrain (e.g., periaquaductal gray (PAG)) and hypothalamus. Of particular note, colocalization of PRV-614 and MC4R-GFP began to become more widespread. Colocalization was still apparent in the RPa, NTS and PVN as well as in several new regions. Double-labeled neurons were concentrated in the rostral ventromedial medulla (e.g., raphe nuclei, NRM, and NGCα), Figure 3, 4, the dorsal brainstem (e.g., NTS), the dorsolateral pons (e.g., Barrington's nucleus and SubC), midbrain (e.g., PAG), and hypothalamus (e.g., PVN, LH). In the PVN (Figure 5), coexpression in all the animals was observed at d5, as well as a large increase in the number of PRV-614-infected neurons, primarily in the ventromedial parvocellular PVN and lateral parvicellular PVN (coexpression in 8% of all PRV-614-infected neurons and 30% of all MC4R neurons in the PVN). The number of singly labeled PRV-614 neurons and the number of colocalized neurons at d5 were increased than that at d4 in the RVMM (coexpression in 12% of all PRV-614-infected neurons and 35% of all MC4R-expressing neurons at d5), in the RPa (coexpression in 12% of all PRV-614-infected neurons and 30% of all MC4R-expressing neurons at d5) and the NTS (coexpression in 10% of all PRV-614-infected neurons and 16% of all MC4R-expressing neurons at d5). The first colocalized neurons were detected in the lateral hypothalamus at d5, and in spite of being small in number, they were observed in all animals (coexpression in 12% of all PRV-614-infected neurons and 20% of all MC4R-expressing neurons at d5).

Areas with PRV-614 staining during phase IV (*n* = 6, 6-7 days postinjection) revealed PRV-614 expression in the majority of hypothalamic sites such as the ventromedial hypothalamus (VMH), lateral hypothalamus (LH), arcuate nucleus (ARC), and preoptic area (POA) as well as motor cortex (M1-M2). Colocalization of PRV-614 and MC4R-GFP was seen in all the same regions as at d5 with small increases in the number of PRV-614-infected neurons in those areas. New regions containing double-labeled PRV-614/MC4R-GFP neurons include the hypothalamus [PVN, DMH, VMH, Arc, retrochiasmatic area (RCh), POA], and forebrain [e.g., motor cortex, Amygdala, central nucleus of amygdale (CeM), bed nucleus of the stria terminalis (BST)]. From d5 to d6-7, the most substantial increase seen in the number of PRV-infected and colocalized cells was in the PVN (coexpression in 23.8% of all PRV-infected neurons and 38.5% of all MC4R-expressing neurons in d 6). The first colocalized neurons were detected in the motor cortex at d6, they were observed in all animals (coexpression in 41.5% of all PRV-infected neurons and 22.7% of all MC4R-expressing neurons).

### Infection of serotonergic neurons following injections of PRV-614 into the kidney

Because a portion of neurons that participate in regulating sympathetic outflow expresses serotonin [46–50], we ascertained whether MC4R-GFP-positive neurons infected after the injection of PRV-614 into the left kidney were serotonergic (5-HT and TPH immunoreactivity positive neurons). Previous studies suggested that TPH-expressing neurons were observed within distinct subdivisions of the CNS, including raphe, VMM, lateral paragigantocellular cell group (LPGi), dorsal raphe (DR), and PVN [51]. In this study, the analysis was limited to the five brain areas containing an appreciable number of PRV-614-infected cells: raphe, VMM, LPGi, DR and PVN. PRV-614/TPH dual-labeled cells and MC4R/PRV-614/TPH triple-labeled cells were present in raphe, VMM, LPGi, and PVN, and the number of dual-labeled cells was much higher than that of triple-labeled cells (Figure 4 and 5). No triple-labeled cells were observed in DR.

## DISCUSSION

To date, this is the first study to explore the neurochemical phenotype of the neurons belonging to the central melanocortinergic efferent pathways innervating the kidneys. PRV-614 retrogradely labeled neurons were found in numerous CNS regions [43, 45, 50, 52], but the proportion of these neurons possessing MC4R-GFP were mainly located in IML of the thoracic spinal cord, the PVN of the hypothalamus, and RPa, NRM and VMM of the brainstem which were believed to be related to the control of energy homeostasis [53].

In this study, we focused principally on those PRV-614-infected areas (neurons) that also express MC4R. Transsynaptic tracing by injection of attenuated PRV-614 into the kidneys in MC4R-GFP transgenic mice, coupled with dual or triple-labeling immunohistochemical techniques, reveals a specific subset of dual or triple-labeled MC4R-GFP/PRV-IR neurons in multiple nuclei at various levels of the CNS. The majority of MC4R-GFP/PRV-614 double-labeled neurons were found in the RPa, NRM and VMM, and with a less extent in the RVLM, A5, and LC. Most of the double-labeled PRV-614/TPH neurons were located in the RPa, NRM and VMM. Accordingly, it seems logical that most of the triple-labeled MC4R-GFP/PRV-614/TPH neurons were located in the same brain regions, i.e., RPa, NRM and VMM. The data presented support previous tracing and neurophysiological investigations that the MC4R signaling pathway, constituting a major signaling system in the control of energy homeostasis [15], is functionally distributed both in the hypothalamus and the brainstem [53], suggesting a broad central melanocortinergic neurons involved in kidney control. The PVN is now commonly recognized as an important brain region exerting prominent influences over endocrine and autonomic regulation [52]. A large number of significant scientific reports demonstrated MC4R in the PVN is important in regulating energy balance and sympathetic activity [54]. The existence of double- and triple-labeled neurons in the PVN is consistent with the proposed role of this brain region.

A considerable amount of literature had demonstrated that serotonin and leptin shared a similar signaling mechanism regulating energy balance and glucose homeostasis *via* actions within the CNS [55]. In the absence of leptin receptors, the renal sympathoexcitatory effects of melanocortin system stimulation were attenuated [38]. 5-HT2CR agonists improved glucose homeostasis *via* increased production and release of endogenous melanocortin agonists, with subsequent activation of sympathetic preganglionic neurons in the IML *via* stimulation of MC4R [56]. These data suggested the major rodent brain regions that contain MC4R and TPH, involved in the descending pathways that control the kidneys.

In conclusion, the present results reveal a hierarchical organization of the descending MC4R neuronal circuits controlling the kidney, thus providing the neuroanatomical support for the role of the MC4R, and to a less extent TPH, in the control of kidneys.

## MATERIALS AND METHODS

### Animal care

The transgenic mice carrying green fluorescent protein (GFP) under the control of the melanocortin-4 receptor promoter coupled with the cytomegalovirus (CMV) immediate early enhancer, were first obtained from Dr. Joel Elmquist (UT Southwestern Medical Center) and then bred to generate male and female mice. Genotyping was performed by PCR as described in previous studied [17, 57]. All mice used were weighted between 25-30 g and maintained in a standard 12h light-dark cycle with *ad libitum* access to food and water in a temperature-controlled room. All studies involving the animals were performed following the National Guides for the Care and Use of Laboratory Animals and approved by the Institutional Animal Care and Use Committee of Tongji Hospital, Tongji Medical College, Huazhong University of Science and Technology University, Wuhan, China.

### Surgical procedures, PRV-614 injection and post-surgical care

To provide a description of the pattern and temporal progression of viral infection resulting from injection of PRV-614 into the kidney, virus was injected into the left kidneys of mice. PRV-614 is a specific trans-synaptic retrograde tracer from axon terminals to the cell nucleus, where replication occurs [2, 27, 58]. Under full anesthesia with isoflurane, the skin overlying the kidney was incised by ventral midline laparotomy to expose the upper pole of the kidney [59, 60]. Sixteen mice received a series of injections with PRV-614 (2×10^8^ pfu/ml in a total of 1 μl per injection at five injection sites per kidney) into the upper pole of the visualized left kidney using a 30-gauge needle connected to a Hamilton syringe (10 μl) under microscopic guidance. After the final injection, the renal surface was rinsed twice with sterile saline-soaked swabs and blotted dry. The kidney was then returned to the abdominal cavity. The abdominal muscle incision was closed with silk sutures, and the skin incision was closed with stainless steel wound clips. The time course of infection was empirically determined by observing the pattern of infection at exactly 3- (*n* = 3), 4- (*n* = 3), 5- (*n* = 4), 6-day (*n* = 3) and 7-day (*n* = 3) survival time. The mice were provided with analgesia with an intramuscular injection of a mixture of ketamine (10 mg/kg) and ketoprofen (3 mg/kg) just before the surgery and every 12 hours subsequently during a post-surgical period of 72 hours. Mice infected with PRV-614 remained asymptomatic until 5-6 days after virus injection, and they were euthanized either before or when they showed apparent traits of illness or distress (i.e. 6-7 days after inoculation).

### Perfusion and fluorescence immunohistochemistry

After a survival time of 3-6 days, mice were deeply anesthetized with an overdose of urethane and perfused with 0.9% saline, which was followed by 4% paraformaldehyde-borate fixative (pH 9.5) *via* the left ventricle. Spinal cord and brain were removed and post-fixed in 4% paraformaldehyde-borate overnight at room temperature and in a 30% sucrose solution for 2 days at 4°C. Post-fixed spinal cord and brains were blocked, and sliced at 30 μm/coronal section on a freezing-stage sledge microtome, and collected into four serially ordered sets of sections through its rostrocaudal extent. Tissue sections were stored at 4°C until they were processed for immunohistochemical visualization.

To determine which PRV-614-immunoreactive (IR) neurons coexpressing MC4R-GFP, distinct fluorophores were used for PRV-614 and GFP. The fluorescence immunohistochemical (IHC) procedure was carried out according to published protocols [40, 61]. In brief, the tissue sections were incubated at 4°C overnight in 0.02M potassium PBS (KPBS; pH7.4), and washed with 0.01M phosphate buffered saline (PBS) (3×10min). Sections were pre-treated for 25 min in 1% sodium borohydride in 0.01M PBS, and washed in 0.01M PBS (3×10min). Sections were blocked for 60min in 0.02M KPBS containing 2% normal donkey serum (Jackson ImmunoResearch, West Grove, PA) and 0.4% Triton X-100 (Sigma, St. Louis, MO), and treated further by washing with 0.01M PBS (3×10min). Sections were incubated with guinea-pig polyclonal antibody against PRV (1:5000; National Institute of Allergy and Infectious Diseases, Bethesda, MD) and a rabbit polyclonal antibody against GFP (1:10,000; Molecular Probes, Eugene, OR) in 0.02M KPBS containing 0.4% Triton X-100 for 48 h at 4°C, with gentle agitation. Sections were then rinsed several times in 0.01M PBS over a 1-h period followed by incubation with Cy3-conjugated donkey anti-guinea pig IgG (1:2000; Jackson ImmunoResearch Laboratories Inc, West Grove, PA) and Alexafluor 488-conjugated donkey anti-rabbit IgG (H+L) (1:1000; Molecular Probes, Eugene, OR) in KPBS containing 0.4% Triton-X-100 for 1h at room temperature. Sections were then rinsed thoroughly in 0.01M PBS. All sections were mounted onto slides, air dried, and cover slipped with mounting media.

Brain sections were also processed for the triple localization of PRV-614, MC4R-GFP, and TPH. The processing of this tissue was similar to that described above. For TPH IHC, first antibody used for immunohistochemical analysis was sheep anti-TPH (1:2000; Chemicon International, Temecula, CA) and second antibody included biotinylated Donkey anti-sheep Ig (H+L) (Lot no.68003, Jackson ImmunoResearch Lab., West Grove, PA) and streptavidin Alexa Fluor 350 conjugate (Lot no.49248A, S-11249, Invitrogen, Molecular Probes, Eugene, OR). After the completion of immunohistochemical processing, the sections were mounted on gelatin-coated slides, dehydrated, cleared, and cover slipped with the use of mounting media. In negative control incubations, the primary antiserums were omitted from the immunohistochemical reaction. This procedure completely eliminated neuronal staining.

### Tissue analysis

The regions in which infected cells were located were defined with reference to the atlases of Franklin KB and Paxinos G [62] and a study by Molander and colleagues [63] that were used to guide the spinal cord tissue analysis. Immunofluorescence photomicroscope was achieved by using the Leica Microsystems (DM400B, Wetzlar, Germany) with a filter set for visualization of Alexafluor 488 (excitation range, 425-525 nm; emission range, 500-600 nm), Cy3 (excitation range, 500-560 nm; emission range, 560-650 nm) and Alexafluor 350 (excitation range, 300-400 nm; emission range, 440-490 nm). PRV-614 infected neurons are identified with red fluorescence, MC4R-expressing neurons by green fluorescence, and TPH-expressing neurons by blue fluorescence. Images were overlaid using Adobe Photoshop, and double-labeled neurons are presented as yellow (red/green) or pink (red/blue). High-magnification analysis was used to determine whether overlapping yellow or pink images were due to colocalization in the same neuron or overlap of independently labeled neurons.
